# Function and Cryo-EM structures of broadly potent bispecific antibodies against multiple SARS-CoV-2 Omicron sublineages

**DOI:** 10.1038/s41392-023-01509-1

**Published:** 2023-07-31

**Authors:** Ping Ren, Yingxia Hu, Lei Peng, Luojia Yang, Kazushi Suzuki, Zhenhao Fang, Meizhu Bai, Liqun Zhou, Yanzhi Feng, Yongji Zou, Yong Xiong, Sidi Chen

**Affiliations:** 1 Department of Genetics, Yale University School of Medicine, New Haven, CT USA; 2 System Biology Institute, Yale University, West Haven, CT USA; 3 Department of Molecular Biophysics and Biochemistry, Yale University, New Haven, CT USA

**Keywords:** Infectious diseases, Biologics, Infectious diseases

**Dear Editor**,

SARS-CoV-2 rapidly evolves during the pandemic^1^ with many variants of concern (VoCs) lineages (Supplementary Fig. S1a). Omicron (B.1.1.529) and its sub-lineages led to multiple infection waves globally.^2^ Omicron subvariants harbored a high number of mutations, especially in the spike (S) glycoprotein, and clustered in the receptor-binding domain (RBD) (Supplementary Fig. S1b, c; Supplemental Discussion). These subvariants drastically decrease the efficacy of current vaccinations and monoclonal antibody therapies.^2^

Among our recently generated Omicron RBD-directed mAbs (MB.02, MB.08, PC.03),^3^ all have strong binding activity against Omicron BA.1, while one of these mAbs, MB.02, maintains high activity against BA.2 (Supplementary Fig. S1d). To enable broad activity against VoCs, we combined Clone13A,^4^ a previously generated SARS-CoV-2 mAb that was potent against SARS-CoV-2 WA-1 and Delta^4^ but had low binding activity against Omicron BA.1 and BA.2 (Supplementary Fig. S1e), with three Omicron mAbs to engineer five bispecific antibodies using classic IgG1 bispecific antibody constructs^5^ (“Methods“) (Fig. 1a). The resultants were named CoV2-0208, CoV2-0203, CoV2-0803, CoV2-0213, and CoV2-0813. SDS-PAGE analysis indicated all purified bispecific antibodies were assembled with the expected size (Fig. 1b, S1f). Thereafter, antibody titration assays were performed by ELISA and showed strong reactivity of these bsAbs (along with S309^6^) to RBDs of SARS-CoV-2 WA-1, Delta, Omicron BA.1 and BA.2 (Supplementary Fig. S2a, b). Meanwhile, our lead bsAbs, CoV2-0213 and CoV2-0813, exhibited strong competition with ACE2 for binding to a range of SARS-CoV-2 RBDs (Supplementary Fig. S3a).

**Fig. 1:**
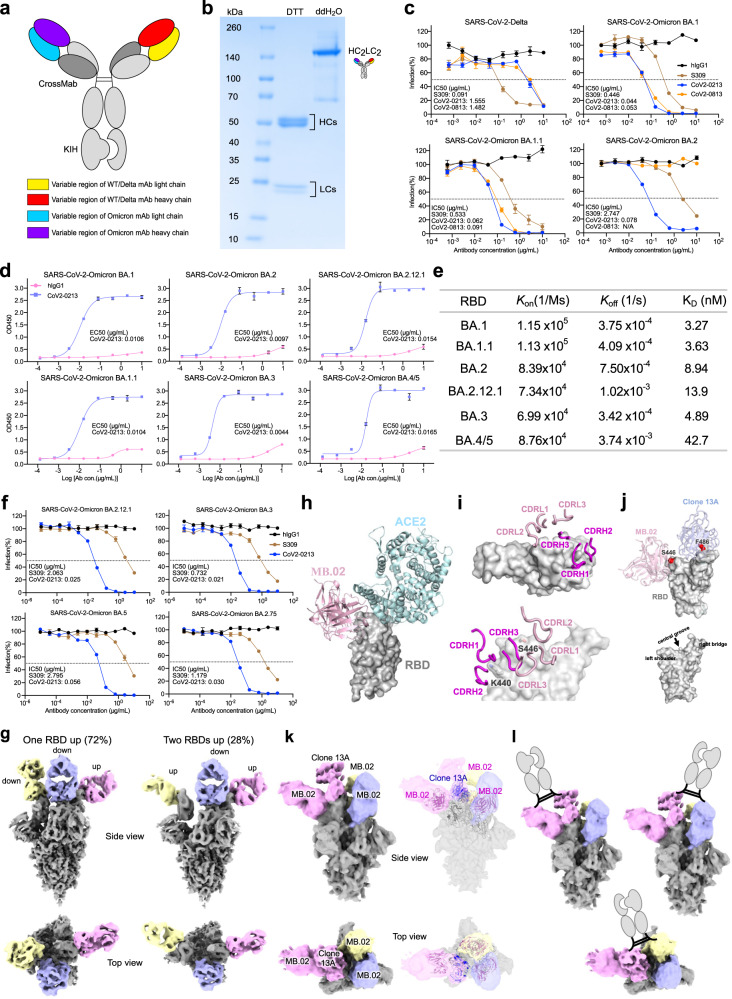
Design, purification, neutralizing activities, and cryo-EM structures of a broadly potent human bispecific antibody CoV2-0213 against circulating Omicron subvariants. **a** A scheme of bsAb design. Antibody domains are colored according to their architecture. **b** Coomassie-stained SDS-PAGE analysis of purified bsAb CoV2-0213. The antibody samples are analyzed under reducing conditions (+DTT) and nonreducing conditions (+ddH_2_O). **c** Neutralization curves of two lead bsAbs with S309 against the pseudotyped virus of SARS-CoV-2 Delta and Omicron sublineages BA.1, BA.1.1, and BA.2. Serial dilutions of bsAb were added to test its neutralizing activity against indicated pseudotyped virus. The IC50 was determined by log (inhibitor) response of nonlinear regression and is displayed as the mean ± s.e.m. **d** ELISA binding curves of CoV2-0213 with RBD proteins of SARS-CoV-2 Omicron sublineages BA.1, BA.1.1, BA.2, BA.2.12.1, BA.3, and BA.4/5. The EC50 was determined by log (agonist) response of nonlinear regression and is displayed as the mean ± s.e.m. **e** The summary statistics of binding affinity of CoV2-0213 to the RBDs of Omicron sublineages as determined by BLI. **f** Neutralization curves of CoV2-0213 and S309 against pseudotyped virus of Omicron sublineages BA.2.12.1, BA.3, BA.5 and BA.2.75. Serial dilutions of bsAb were added to test its neutralizing activity against indicated pseudovirus. The IC50 was determined by log (inhibitor) response of nonlinear regression and is displayed as the mean ± s.e.m. **g** Cryo-EM structures of MB.02 Fab fragment in complex with spike trimer in two different conformations, with one RBD up (left) or two RBDs up (right). Fab molecules are shown in different colors, and the spike is shown as dark gray. The corresponding particle distribution of each spike trimer conformation is shown. **h** Overlay of the structures of human ACE2 (PDB 7wpb) and MB.02 Fab onto the same spike RBD by superimposing the RBD regions. **i** The binding conformations of the three CDRH and the three CDRL loops of MB.02 Fab on spike RBD. Upper panel, top view; lower panel, side view. The omicron BA.1.1.529 mutation N440K and G446S, which is located at the MB.02 binding interface and inserted between CDRH1 and CDRH2 loops of MB.02 Fab, is shown in stick representation. The nomenclature of the top surface of spike RBD is illustrated in the middle inset. **j** Overlay of the structures of the humanized clone 2 (Clone 13A) and MB.02 Fab fragments on the same spike RBD. S446 at the MB.02-bound interface of Omicron BA.1/3 spike RBD, and F486 at the Clone 13A-bound interface of WT and Omicron BA.1/2/3 spike RBD, are shown as red spheres. The nomenclature of the top surface of spike RBD is illustrated in the lower panel. **k** Cryo-EM reconstruction (left panel) and fitted models (right panel) of the CoV2-0213 antibody-bound Omicron BA.5 spike trimer with one RBD up. Fab molecules are shown in different colors, and the spike is shown in dark gray. The up RBD has both MB.02 and Clone 13A Fab fragments bound at distinct interfaces. The other two RBDs in down conformation have the MB.02 Fab bound. The models of the Fab variable domains (MB.02, pink; Clone 13A, blue; RBD, black) are fitted into the 3D reconstruction. **l** Cartoon illustrations of the possible bivalent binding of one CoV2-0213bsAb onto a single or two adjacent spike RBDs in the same S trimer. The Fc fragment and hinge region of the IgG is shown in the cartoon

Next, we performed neutralization assays with pseudoviruses (Supplementary Fig. S3b), a widely used assay by the field that is well-correlated with authentic virus assays.^7–10^ S309 retained neutralization activity against BA.1 and BA.1.1 but dropped 30-fold against BA.2 (Fig. 1c). In contrast, CoV2-0213 displayed broad-spectrum neutralization to a range of circulating Omicron subvariants (Fig. 1c, f). Specifically, CoV2-0213 remained potent in neutralizing seven of the Omicron sublineages, BA.1, BA.1.1, BA.2, BA.2.12.1, BA.3, BA.4/5, and BA.2.75 with IC_50_ values of 0.044, 0,062, 0.078, 0.025, 0.021, 0.056, and 0.030 µg/mL, respectively (Fig. 1c, f). CoV2-0213 was ~10× more potent than S309 in neutralization against BA.1 and BA.1.1 and ~78× more potent than S309 against BA.2 (Fig. 1c). Meanwhile, two other bispecific antibodies exhibited potent Omicron-specific neutralization (Fig. 1c). CoV2-0203 showed high potency in neutralizing three of Omicron sublineages, although it showed relatively weak neutralization (1.5-fold) compared with CoV2-0213 (Supplementary Fig. S4b). CoV2-0208 showed high potency against BA.1 and BA.1.1, but its activity against BA.2 is on par with S309 (Supplementary Fig. S4b). Both CoV2-0208 and CoV2-0203 lost neutralization against Delta. CoV2-0213 has strong neutralization potency against Omicron BA.1, BA.1.1, and BA.2 and maintains reasonable activity against Delta.

To evaluate antibody cross-reactivity, we tested eight human coronaviruses RBD proteins, including six Omicron sublineages and two β-coronaviruses. The results showed CoV2-0213 has a broad and strong binding activity to all assayed Omicron RBDs (Fig. 1d) but weak binding to β-coronaviruses RBDs (Supplementary Fig. S2c). Biolayer interferometry results revealed CoV2-0213 displayed high affinity to Omicron BA.1 (*K*_d_ = 3.27 nM), BA.1.1 (*K*_d_ = 3.63 nM), BA.2 (*K*_d_ = 8.94 nM),_BA.2.12.1 (*K*_d_ = 13.9 nM), BA.3 (*K*_d_ = 4.89 nM) and BA.4/5 (*K*_d_ = 42.7 nM), respectively (Fig. 1e, Supplementary S5a, b).

We then determined the cryo-EM structures of MB.02 Fab in complex with Omicron BA.1 spike trimer at ~3.2 Å resolution (Supplementary Table S1). Two major S trimer conformation states were detected, one with one RBD up (72%) and the other with two RBDs up (28%) (Fig. 1g, Supplementary Fig. S6a, Fig. S7a), indicating that MB.02 bound to up or down conformation of RBD regardless of neighboring RBD conformation. In both conformations, the S trimer was bound with three Fabs, one per RBD, suggesting binding of MB.02 Fab is more flexible, especially in up conformation (Supplementary Fig. S6b). MB.02 mainly contacted a flexible loop region at the left shoulder region of the spike, and no overlap with the ACE2-binding interface (Fig. 1h). All six CDRs of MB.02 involve in RBD interactions (Fig. 1i). MB.02 binding interface contained two key residues (K440 and S446), with K440 contacting CDRH1 and CDRH2 of MB.02, and S446 interacting with CDRL2 loop (Fig. 1i, lower panel), indicating MB.02 has distinct binding epitopes to interact with spike compared with clinically authorized SARS-CoV-2 mAbs (Supplementary Fig. S8). Furthermore, we analyzed spike mutations in BA.2.12.1, BA.3, and BA.4/5 that were directly located at the CoV2-0213 binding interface. Residue S446 was important for MB.02 binding (Fig. 1j), was present in BA.3 but not in BA.2.12.1 and BA.4/5, while F486V may disrupt Clone 13A interaction, was present in BA.4/5 only (Fig. 1j). These differences may explain the enhanced binding affinity of CoV2-0213 to different Omicron subvariants, although other spike mutations may also have indirect allosteric effects on spike conformation at CoV2-0213 binding interface. To confirm the structural result and evaluate possible antibody evasion, we performed a neutralization assay with K440A/S446A/V486A Omicron BA.1 pseudovirus. The result demonstrated that triple-alanine BA.1 mutant could result in strong resistance to CoV2-0213, indicating the above residues are key epitopes of CoV2-0213, consistent with our structural findings (Supplementary Fig. S4a).

Taken together, CoV2-0213 exhibited significantly enhanced activities to a wide range of assayed Omicron subvariants compared to its parental mAbs,^3^ which prompts us to investigate its distinct mechanism of action. One Fab arm of CoV2-0213, Clone 13A,^4^ mainly interacts with a right ridge of the spike and would not lead to any steric clash with a bound MB.02, suggesting both arms of CoV2-0213 could target the same spike (Fig. 1j). To further investigate how CoV2-0213 binds to spike, we determined the cryo-EM structure of CoV2-0213 in complex with Omicron BA.5 subvariant. Among the cryo-EM particles we collected, only one spike conformation with one RBD up was detected (Fig. 1k, Supplementary Fig. S7b). Thereafter, we identified a subset (~24%) of particles with density for two Fab fragments on the same RBD in up conformation and one Fab fragment each for the other two RBDs in down conformation (Fig. 1k, left panel) at a resolution of 7.7 Å (unmasked and unsharpened) (Supplementary Table S1). The density for Fab-bound up-RBD fitted well with the overlaid model of spike with MB.02 and Clone 13A Fab fragments,^4^ while the density of Fab-bound down RBDs matched with the model of MB.02 Fab-bound RBD (Fig. 1k, right panel). This suggests three MB.02 and one Clone 13A bound to the same S trimer, potentially from three CoV2-0213, with one having both Fab arms bound. MB.02 could bind to RBD regardless of the bound and neighboring RBD conformations. However, Clone 13A could only bind to either an up RBD or a down RBD with a neighboring up RBD due to spatial clash with a neighboring down RBD.^4^ From the cryo-EM data, we only observed MB.02 and Clone 13A simultaneously bind on an up RBD, but theoretically, they could also bind on a down RBD with a neighboring up RBD in a trimeric spike (Supplementary Fig. S6c, d). Considering the flexible nature of the hinge region of an IgG, it is possible that the two Fab arms of a CoV2-0213 can target one single spike or two adjacent ones in the same trimer (Fig. 1l). Alternative interpretation is four Fabs from four different CoV2-0213 bound to spike trimer. Regardless of the binding modes, the cryo-EM data revealed epitope co-engagement mechanism and supported enhanced affinity of Omicron spike and neutralization activity by CoV2-0213.

## Supplementary information

Supplement - using STTT template

## Data Availability

All relevant primary data of this study are included in the manuscript, its supplements, and the public repository (CryoEM structure data accessions listed in Table S1). Additional data requests can be made to the corresponding authors.

## Source data

original_source_data_stats_v7

## References

[1] Acuti Martellucci C (2020). SARS-CoV-2 pandemic: an overview. Adv. Biol. Regul..

[2] Liu L (2022). Striking antibody evasion manifested by the Omicron variant of SARS-CoV-2. Nature.

[3] Ren P (2023). RAMIHM generates fully human monoclonal antibodies by rapid mRNA immunization of humanized mice and BCR-seq. Cell Chem. Biol..

[4] Peng L (2022). Monospecific and bispecific monoclonal SARS-CoV-2 neutralizing antibodies that maintain potency against B.1.617. Nat. Commun..

[5] Schaefer W (2011). Immunoglobulin domain crossover as a generic approach for the production of bispecific IgG antibodies. Proc. Natl Acad. Sci. USA.

[6] Gupta A (2021). Early Treatment for Covid-19 with SARS-CoV-2 Neutralizing Antibody Sotrovimab. N. Engl. J. Med..

[7] Bewley KR (2021). Quantification of SARS-CoV-2 neutralizing antibody by wild-type plaque reduction neutralization, microneutralization and pseudotyped virus neutralization assays. Nat. Protoc..

[8] Garcia-Beltran WF (2022). mRNA-based COVID-19 vaccine boosters induce neutralizing immunity against SARS-CoV-2 Omicron variant. Cell.

[9] Liu L (2020). Potent neutralizing antibodies against multiple epitopes on SARS-CoV-2 spike. Nature.

[10] Nie J (2020). Quantification of SARS-CoV-2 neutralizing antibody by a pseudotyped virus-based assay. Nat. Protoc..

