# Supplementary material for: Function and Cryo-EM structures of broadly potent bispecific antibodies against multiple SARS-CoV-2 Omicron sublineages
Source: Signal Transduct Target Ther. 2023 Jul 31;8:281. doi: 10.1038/s41392-023-01509-1 (PMC10387464; doi:10.1038/s41392-023-01509-1)
Supplement: Supplementary file 1 — Supplement - using STTT template [file 41392_2023_1509_MOESM1_ESM.pdf]

Supplementary materials for

Function and Cryo-EM structures of broadly potent bispecific antibodies against multiple SARS-CoV-2 Omicron sublineages

Ping Ren [1,2,*], Yingxia Hu [3,*], Lei Peng [1,2,*], Luojia Yang [1,2], Kazushi Suzuki [1,2], Zhenhao Fang [1,2], Meizhu Bai [1,2], Liqun Zhou [1,2], Yanzhi Feng [1,2], Yongji Zou [1,2], Yong Xiong [3,@], and Sidi Chen [1,2,@]

1. Department of Genetics, Yale University School of Medicine, New Haven, CT, USA
2. System Biology Institute, Yale University, West Haven, CT, USA
3. Department of Molecular Biophysics and Biochemistry, Yale University, New Haven, CT, USA

* Co-first authors.

@ Correspondence: SC (sidi.chen@yale.edu), YX (yong.xiong@yale.edu)

Materials and Methods

Cloning and expression of SARS-CoV2-specific human bispecific antibodies

Bispecific antibodies were cloned and expressed using methods similar to those previously described [1], utilizing the Fab regions from four of our previously developed fully human or largely humanized monoclonal antibodies, Clones MB.02, MB.08, PC.03[2] and Clone 13A[1]. In brief, indicated mAb variable regions for each bispecific antibody were amplified and subcloned into separate mammalian expression vectors using Gibson assembly[1]. To express recombinant bispecific antibodies, four expression vectors were transiently transfected into Expi293 cells using ExpiFectamine 293 transfection kit according to the manufacturer's protocol (ThermoFisher). Antibody containing cell culture supernatants were harvested after 5 days of cultivation in shake flasks, then secreted bispecific antibodies were collected and purified by affinity chromatography using rProtein A Sepharose Fast Flow beads (Cytiva, Cat: #17127901). Purified bispecific antibodies were inspected using SDS-PAGE and stored at -80°C after further usage.

SARS-CoV-2 pseudotyped virus generation and neutralization assay

The SARS-CoV-2 pseudotyped virus was produced as previously described[2]. Briefly, pseudotyped virus containing cell culture supernatant was harvested after 2 days of co-

transfection of HEK-293T cells with a spike-expressing plasmid and env-deficient HIV-1 backbone vectors, then clarified by centrifugation and stored at -80℃ after further usage. To determine the neutralizing activity of the bispecific antibody, serially diluted antibodies were incubated with pseudotyped virus at 37℃ for 1 hour, then co-cultured with HEK-293T-hACE2 cells for overnight. Finally, the signal was evaluated after 48 hours by detection of GFP expression in the HEK-293T-hACE2 cells using Attune NxT Acoustic Focusing Cytometer (ThermoFisher) or BD Symphony Flow Cytometry.

Antibody binding quantification and ACE2 competition assay

The binding of bispecific antibodies was quantified by ELISA and ACE2 competition assays, as previously described[2]. The recombinant MERS-CoV RBD (Cat. No. 50-201-9463), SARS-CoV RBD (Cat. No. 50-196-4017), SARS-CoV-2 RBD wild type (WA-1) (Cat. No. 40592-V08B), SARS-CoV-2 Delta RBD (Cat. No.40592-V08H90), SARS-CoV-2 Omicron BA.1 RBD (Cat. No.40592-V08H121), SARS-CoV-2 Omicron BA.1.1 RBD (Cat. No. SPD-C522j-100ug), SARS-CoV-2 Omicron BA.2 RBD (Cat. No.SPD-C522G-100ug), SARS-CoV-2 Omicron BA.2.12.1 RBD (Cat. No. SPD-C522Q-100ug), SARS-CoV-2 Omicron BA.3 RBD (Cat. No. SPD-C522I-100ug), SARS-CoV-2 Omicron BA.4/5 RBD (Cat. No. SPD-C522R-100ug) used in ELISA quantification were purchased from Thermo, Sino Biological and AcroBiosystems, respectively[2]. The ACE2 (Cat. No.10108-H08H) used in the ACE2 competition assay was purchased from Sino Biological [1].

Bispecific antibody binding affinity measurement

The binding affinity of antibodies with RBD was performed previously by Octet RED96e (ForteBio) using bio-layer interferometry (BLI). 25ng/µl of purified bispecific antibody was immobilized onto AHC biosensors (ForteBio) with recombinant Omicron RBDs acting as the analyte with serial dilutions. Kd values were calculated using Data Analysis HT 10 (ForteBio) with a 1:1 Langmuir binding model.

Cryo-EM sample preparation and data collection

The omicron B.1.1.529 spike trimer (Sino Biological Cat: 40589-V08H26) at a final concentration of 0.6 mg/mL was mixed with MB.02 Fab at a molar ratio of 1:1.5 on ice for 30mins. The omicron BA.5 spike trimer (ACROBiosystems SPN-C522e-50ug) at a final concentration of 0.6 mg/mL was mixed with the bispecific antibody 0213 at a molar ratio of 1:1 on ice for 30mins. Then 3 µl of the mixture was applied to a Quantifoil-Au-2/1-3C grid (Quantifoil) pretreated by glow-discharging at 20 mA for 1 min. The grid was blotted at 18 °C with 100% humidity and plunge-frozen in liquid ethane using FEI Vitrobot Mark IV (Thermo Fisher). The grids were stored in liquid nitrogen until data collection.

Images were acquired on an FEI Titan Krios electron microscope (Thermo Fisher) equipped with a Gatan K3 Summit direct detector in super-resolution mode, at a calibrated magnification of 81,000× with the physical pixel size corresponding to 1.07

Å. Detailed data collection statistics for the Fab-spike trimer complexes are shown in a supplemental table. Automated data collection was performed using SerialEM [3].

Cryo-EM data processing

A total of 5373 movie series were collected for the complex of MB.02 Fab with omicron BA.1 S trimer. A total of 2408 movie series were collected for the complex of CoV2-0213 bsAb with the omicron BA.5 S trimer. Motion correction of the micrographs was carried out using RELION [4] and contrast transfer function (CTF) estimation was calculated using CTFFIND4 [5]. Particles were picked automatically by crYOLO [6], followed by 2D and 3D classifications without imposing symmetry. The 3D classes with different S trimer conformations were then processed separately by consensus 3D refinement and CTF refinement. For each state of the complex, local masked 3D classification without image alignment was performed focusing on one Fab-RBD region, and the best class of particles was selected for consensus refinement of the whole complex. For the complex of CoV2-0213 bsAb with the omicron BA.5 S trimer, repeated consensus 3D refinement and subsequent local masked 3D classification without image alignment, focusing on the up RBD with the two associated Fabs, were performed to identify the class of S trimers with both MB.02 and Clone 13A Fabs bound to the same up RBD. Subsequently, multibody refinement was performed as described above for the rigid body containing the focused region. The 3D reconstruction of the other Fab-RBD regions were obtained with the same procedure. The final resolution of each reconstruction was determined based on the Fourier shell correlation (FSC) cutoff at 0.143 between the two half maps [7]. The final map of each body was corrected for K3 detector modulation and sharpened by a negative B-factor estimated by RELION [8], and then merged in Chimera for deposition. The local resolution estimation of each cryo-EM map is calculated by RELION [4].

Model building and refinement

The structure of SARS-CoV-2 omicron BA.1 spike trimer (PDB 7qo7) was used as an initial model and docked into the spike trimer portion of the cryo-EM maps using Chimera [9]. The initial model of MB.02 Fab was generated by homology modeling using SWISS-MODEL [10], and then docked into the Fab portions of the cryo-EM maps using Chimera [9]. The initial models were subsequently manually rebuilt in COOT [11], followed by real-space refinement in PHENIX [12]. The final models with good geometry and fit to the map were validated using the comprehensive cryo-EM validation tool implemented in PHENIX [13]. All structural figures were generated using PyMol (http://www.pymol.org/) and ChimeraX [14].

Supplementary Text

The SARS-CoV-2 pathogen rapidly disseminates globally, with a constantly evolving landscape in the pandemic [15]. To date, the COVID-19 pandemic has infected nearly 600 million confirmed individuals and caused over 6.4 million deaths (https://www.worldometers.info/coronavirus/). One of the most concerning features of the virus is that the pathogen SARS-CoV-2 continues to evolve. Numerous mutant lineages emerged, some became dominant while some diminished, leading to multiple waves across the world. The Omicron variant (B.1.1.529), initially reported in South Africa in late 2021, rapidly became the predominant variant circulating in many countries and led the fourth pandemic wave globally [16]. As compared with the genome sequences of ancestral variants of concerns (VOCs), the Omicron lineage has harbored a high number of genomic mutations, especially in the spike (S) glycoprotein and clustered in the receptor-binding domain (RBD). These mutations drastically decrease the efficacy of currently available vaccines and FDA-approved or emergency-use authorized (EUA) monoclonal antibody-based therapies [16-22]. Therefore, it is critical to rapidly develop countermeasures such as new antibodies against these Omicron sublineages. As echoed in recently authorized bi-valent vaccines (Pfizer/BioNTech) and Moderna), it has gain increasing traction to target both Omicron and ancestral lineages that are substantially different, for which a bispecific antibody can achieve with one arm targeting Omicron and the other targeting ancestral lineage.

As of today, the original Omicron lineage has evolved into multiple distinct sub-lineages, such as BA.1, BA.2, BA.3, and BA.4/BA.5 according to WHO weekly epidemiological update[23-25]. BA.1.1 (a subvariant of BA.1) has an additional R346K mutation and caused large regional outbreaks in Canada with BA.1 during the first quarter of 2022[26]. BA.2.12.1 (a subvariant of BA.2), which carried two additional alterations (L452 and S704) on top of BA.2, has emerged and once become the dominant variant in the US and certain other regions [27]. In addition, BA.4/BA.5, which shared an identical sequence of the spike protein, contained additional mutations (Del69-70, L452R, F486V, and R493Q) compared to BA.2, and become dominant in several major regions of the world, including the US and China[28]. These phenomena indicate rapid dynamics of the viral evolution, leading to substantial changes in infectivity, antigenic escape, reduction of vaccine efficacy, and increased possibility of repeat infections[19,20,27,29,30]. Although vaccines are available, therapeutic antibodies remain critical for infected and especially hospitalized patients. However, few clinical monoclonal antibodies can retain substantial activities against all Omicron sublineages [27], urging for more therapeutic options[28,31]. Therefore, it is critical to rapidly develop countermeasures such as new antibodies against these sublineages.

In this correspondence study, we, therefore, set out to develop new candidates of monoclonal therapeutic antibodies that can counter multiple Omicron sub-lineages. To avoid potential immune escape and infection by SARS-CoV-2 mutational variants, cocktail strategies (i.e., administration of two human monoclonal antibodies) were previously designed and reported, which had been demonstrated that could largely

increase efficacy and maximally prevent viral escape [32-34]. However, the cocktail strategy requires the production of multiple molecules. On the contrary, the bispecific antibody (bsAb) strategy can simultaneously target two different antigens or antigenic sites according to its structural design. In addition, bsAb can benefit from the synergistic effects of the two binder Fab arms [35]. An effective dual-binder bsAb can also save the manufacturing process cost by half as compared to a cocktail of two mono-specific mAbs, which can be substantial in translational and clinical stage development. Furthermore, the dual-targeting concept has already been successfully studied as an effective strategy in the treatment of cancer and inflammatory disorders [36-38]. In addition, several studies have illustrated that bispecific antibodies could potently enhance breadth and potency than parental monoclonal antibodies [39-41]. Due to extensive mutations in the spike protein, Omicron sublineages are drastically different from the variants in the ancestral lineages (e.g., wildtype, WT, Wuhan-1, WA-1) and earlier variants (such as Alpha variant, Beta variant, Delta variant).

We have generated five distinct SARS-CoV-2-targeting nbsAbs (CoV2-0208, CoV2-0203, CoV2-0803, CoV2-0213, and CoV2-0813), and evaluated their functional properties against the ancestral and Omicron (sub)lineages. We included the clinically relevant mAb S309 (Sotrovimab [42]) in the experiment, which has been in human use under Food and Drug Administration (FDA) issued emergency use authorization (EUA) in the US, and similarly in Europe, UK, Japan, and Australia. The mAb S309 retained neutralization activity against both Omicron BA.1 and BA.1.1 (BA.1+R346 mutation) with half-maximum inhibitory concentration (IC_{50}) values of 0.44 and 0.53 μg/mL, respectively. However, its neutralization activity against Omicron BA.2 dropped 30-fold to an IC_{50} value of 2.75μg/mL. Of note, the EUA of S309/Sotrovimab was recently withdrawn by FDA in March 2022 due to the high prevalence of the BA.2 variant in many states in the US.

To characterize our bispecific antibody candidates for their reactivity against multiple SARS-CoV-2 lineages, we performed a series of assays, including ELISA, Biolayer interferometry (BLI), and neutralization. To better understand the mechanism of action of the bsAb CoV-0213, we performed cryo-electron microscopy (cryo-EM) studies to solve the three-dimensional (3D) structures of the bsAb and its major Fab arm (MB.02) in complexes with the Omicron spike variants (we previously reported the cryo-EM structure of the other Fab arm, 13A in complex with WT spike[1]). We first sought to map the molecular basis for the broad specificity of MB.02 and its interaction with the RBD epitope and determined the cryo-EM structures of MB.02 Fab in complex with the ectodomain of Omicron BA.1 spike trimer (S trimer) at ~3.2 Å resolution. To further investigate how CoV2-0213 bsAb binds to the spike antigen, we determined the cryo-EM structure of CoV2-0213 bsAb in complex with the recently emerged and prevalent BA.5 Omicron subvariant. The omicron BA.1 spike RBD maintains an overall architecture similar to that of the WT spike from the ancestorial lineage, with minor local conformational changes primarily at the mutation sites.

The lead bispecific antibody, CoV2-0213, exhibited significantly or even enhanced binding affinity and neutralizing efficacy to a wide range of assayed Omicron subvariants compared to its parental monoclonal antibodies[2], suggesting its promise as a potent and more cost-effective candidate as compared to using two single mAbs. To further investigate how CoV2-0213 bsAb binds to the spike antigen, we determined the cryo-EM structure of CoV2-0213 bsAb in complex with the recently emerged and prevalent BA.5 Omicron subvariant (CoV-0213::BA.5 Complex). The flexible Fc region could not be visualized in the cryo-EM reconstruction and therefore we could not identify which bound MB.02 Fab and Clone 13A Fab were from the same CoV2-0213 bsAb. This structure showed that three MB.02 and one Clone 13A Fabs bound to the same S trimer, potentially from three 0213 bsAbs with one having both Fab arms bound. Considering the flexible nature of the hinge region of an IgG, it is possible that the two Fab arms of a CoV2-0213 bsAb can target one single spike RBD or two adjacent ones in the same trimer simultaneously. It is also possible the observed four bound-Fab fragments are from four different CoV2-0213 bsAbs. Regardless of which mode the bsAb binds to the spike RBD, our observation explains the enhanced engagement of the Omicron spike and neutralization effect by CoV2-0213.

Our lead nbsAb, CoV2-0213, exhibited potent and broad-spectrum activities ability to all major Omicron sublineages (BA.1, BA.1.1, BA.2, BA.2.12.1, BA.3 BA.4/BA.5, and BA.2.75). Our cryo-EM structure demonstrated that the two Fabs arms of CoV2-0213 can fully engage the same SARS-CoV-2 spiker trimer simultaneously. Furthermore, Omicron BA.1 triple-alanine mutant abolished neutralization to CoV-0213, indicating K440, S446, and V486 are the key residues that retained the neutralizing activities of CoV2-0213.

In summary, we have generated five distinct SARS-CoV-2-targeting human nbsAbs, and the lead nbsAb, CoV2-0213, exhibited potent and broad-spectrum activity's ability to a range of SARS-CoV-2 VoCs, including various Omicron sublineages and ancestral lineages. Cryo-EM structure demonstrated that the two Fabs arms of CoV2-0213 can fully engage the same RBD simultaneously. CoV2-0213 is primarily human and ready for translational testing.

<u>Limitation of the study:</u> It is worth noting that the pandemic evolves quickly, and new variants constantly emerge. In the past two months, recently circulating variants include variants such as BQ.1.1 and XBB.1 and their sublineages, which display significant growth advantages over BA.2.75 and BA.5. It is important to emphasize that BQ.1.1 and XBB.1 sublineages are now completely resistant to both clinically authorized LY-CoV1404 and Evusheld (COV2-2196 + COV2-2130). From structural analysis, CoV2-0213 likely lost neutralizing activity against BQ.1.1 and XBB.1 due to convergent evolution of Omicron RBD (R346T+K444T/V445P/S446G+F486V/S) (**Supplementary Fig. S4c**). V445P is found in XBB and XBB.1, mutating (residue 445) valine to proline that caused steric hindrance and additional hydrogen bond formation that impaired the interaction with residue Y50 of MB.02. Similarly, K444T and S446G

are presented in BQ.1 and BQ.1.1, mutating (residue 444) lysine to threonine that decreased the interaction with MB.02 due to shorter and uncharged side chain, and mutating (residue 446) serine to glycine that abrogated the binding of this residue with MB.02 directed to this site. Thus, either mutation would dramatically impair the neutralizing activities of MB.02. Furthermore, F486 mutation was observed in both BQ.1.1 (F486V) and XBB.1(F486S), which would disrupt the binding of Clone13A to the RBD domain.

Furthermore, the antibody neutralization breadth is not only governed by the biochemical properties but also depends on the virus mutation pattern and evolution. BQ.1 and XBB subvariants, the most recent circulation of Omicron subvariants to date (April – May 2023), are carrying those convergent mutations in the spike that made them exhibit far greater antibody resistance than earlier variants. These variants and lineages escape almost all of the currently available antibodies including clinically emergency-authorized ones, as well as COV2-0213. RBD mutations can render CoV2-0213 less effective against BQ.1.1 or XBB.1. Therefore, this challenge continues to call for rapid development of diverse repertoire of lineage-specific and broadly neutralizing antibodies. Thus, future studies may go through additional and advanced antibody engineering to identify and develop further enhanced high affinity neutralizing bispecific antibodies against these newly emerged lineages such as XBB.

Supplementary references

1 Peng, L. *et al.* Monospecific and bispecific monoclonal SARS-CoV-2 neutralizing antibodies that maintain potency against B.1.617. *Nat. Commun.* **13**, 1638 (2022).

2 Ren, P. *et al.* RAMIHM generates fully human monoclonal antibodies by rapid mRNA immunization of humanized mice and BCR-seq. *Cell Chem. Biol.* **30**, 85-96 e86 (2023).

3 Mastronarde, D. N. Automated electron microscope tomography using robust prediction of specimen movements. *J. Struct. Biol.* **152**, 36-51 (2005).

4 Kimanius, D., Dong, L., Sharov, G., Nakane, T. & Scheres, S. H. W. New tools for automated cryo-EM single-particle analysis in RELION-4.0. *Biochem. J.* **478**, 4169-4185 (2021).

5 Rohou, A. & Grigorieff, N. CTFFIND4: Fast and accurate defocus estimation from electron micrographs. *J. Struct. Biol.* **192**, 216-221 (2015).

6 Wagner, T. *et al.* SPHIRE-crYOLO is a fast and accurate fully automated particle picker for cryo-EM. *Commun. Biol.* **2**, 218 (2019).

7 Scheres, S. H. & Chen, S. Prevention of overfitting in cryo-EM structure determination. *Nat. Methods* **9**, 853-854 (2012).

8 Rosenthal, P. B. & Henderson, R. Optimal determination of particle orientation, absolute hand, and contrast loss in single-particle electron cryomicroscopy. *J. Mol. Biol.* **333**, 721-745 (2003).

9 Pettersen, E. F. *et al.* UCSF Chimera--a visualization system for exploratory research and analysis. *J. Comput. Chem.* **25**, 1605-1612 (2004).

10 Waterhouse, A. *et al.* SWISS-MODEL: homology modelling of protein structures and complexes. *Nucleic Acids Res.* **46**, W296-W303 (2018).

11 Emsley, P., Lohkamp, B., Scott, W. G. & Cowtan, K. Features and development of Coot. *Acta Crystallogr. D Biol. Crystallogr.* **66**, 486-501 (2010).

12 Afonine, P. V. *et al.* Real-space refinement in PHENIX for cryo-EM and crystallography. *Acta Crystallogr. D Struct. Biol.* **74**, 531-544 (2018).

13 Afonine, P. V. *et al.* New tools for the analysis and validation of cryo-EM maps and atomic models. *Acta Crystallogr. D Struct. Biol.* **74**, 814-840 (2018).

14 Pettersen, E. F. *et al.* UCSF ChimeraX: Structure visualization for researchers, educators, and developers. *Protein Sci* **30**, 70-82 (2021).

15 Acuti Martellucci, C. *et al.* SARS-CoV-2 pandemic: An overview. *Adv. Biol. Regul.* **77**, 100736 (2020).

16 Liu, L. *et al.* Striking antibody evasion manifested by the Omicron variant of SARS-CoV-2. *Nature* **602**, 676-681 (2022).

17 Cao, Y. *et al.* Omicron escapes the majority of existing SARS-CoV-2 neutralizing antibodies. *Nature* **602**, 657-663 (2022).

18 Cele, S. *et al.* Omicron extensively but incompletely escapes Pfizer BNT162b2 neutralization. *Nature* **602**, 654-656 (2022).

19 Iketani, S. *et al.* Antibody evasion properties of SARS-CoV-2 Omicron sublineages. *Nature* **604,** 553-556 (2022).

20 Planas, D. *et al.* Considerable escape of SARS-CoV-2 Omicron to antibody neutralization. *Nature* **602**, 671-675 (2022).

21 Rossler, A., Riepler, L., Bante, D., von Laer, D. & Kimpel, J. SARS-CoV-2 Omicron Variant Neutralization in Serum from Vaccinated and Convalescent Persons. *N. Engl. J. Med.* **386**, 698-700 (2022).

22 Zhou, T. *et al.* Structural basis for potent antibody neutralization of SARS-CoV-2 variants including B.1.1.529. *Science* **376,** eabn8897 (2022).

23 Evans, J. P. *et al.* Neutralization of the SARS-CoV-2 Deltacron and BA.3 Variants. *N. Engl. J. Med.* **386**, 2340-2342 (2022).

24 Hachmann, N. P. *et al.* Neutralization Escape by SARS-CoV-2 Omicron Subvariants BA.2.12.1, BA.4, and BA.5. *N. Engl. J. Med.* **387**, 86-88 (2022).

25 Yu, J. *et al.* Neutralization of the SARS-CoV-2 Omicron BA.1 and BA.2 Variants. *N. Engl. J. Med.* **386**, 1579-1580 (2022).

26 Brown, P. E. *et al.* Omicron BA.1/1.1 SARS-CoV-2 Infection among Vaccinated Canadian Adults. *N. Engl. J. Med.* **386**, 2337-2339 (2022).

27 Wang, Q. *et al.* Antibody evasion by SARS-CoV-2 Omicron subvariants BA.2.12.1, BA.4, & BA.5. *Nature* **608,** 603-608 (2022).

28 Takashita, E. *et al.* Efficacy of Antibodies and Antiviral Drugs against Omicron BA.2.12.1, BA.4, and BA.5 Subvariants. *N. Engl. J. Med.* **387,** 468-470 (2022).

29 Tuekprakhon, A. *et al.* Antibody escape of SARS-CoV-2 Omicron BA.4 and BA.5 from vaccine and BA.1 serum. *Cell* **185**, 2422-2433 e2413 (2022).

30 Cao, Y. *et al.* BA.2.12.1, BA.4 and BA.5 escape antibodies elicited by Omicron infection. *Nature* **608,** 593-602 (2022).

31 Vangeel, L. *et al.* Remdesivir, Molnupiravir and Nirmatrelvir remain active against SARS-CoV-2 Omicron and other variants of concern. *Antiviral Res.* **198**, 105252 (2022).

32 Baum, A. *et al.* REGN-COV2 antibodies prevent and treat SARS-CoV-2 infection in rhesus macaques and hamsters. *Science* **370**, 1110-1115 (2020).

33 Baum, A. *et al.* Antibody cocktail to SARS-CoV-2 spike protein prevents rapid mutational escape seen with individual antibodies. *Science* **369**, 1014-1018 (2020).

34 Wang, N. *et al.* Structure-based development of human antibody cocktails against SARS-CoV-2. *Cell Res.* **31**, 101-103 (2021).

35 Li, C. *et al.* Broad neutralization of SARS-CoV-2 variants by an inhalable bispecific single-domain antibody. *Cell* **185**, 1389-1401 e1318 (2022).

36 Demanet, C., Brissinck, J., De Jonge, J. & Thielemans, K. Bispecific antibody-mediated immunotherapy of the BCL1 lymphoma: increased efficacy with multiple injections and CD28-induced costimulation. *Blood* **87**, 4390-4398 (1996).

37 Li, J. F., Niu, Y. Y., Xing, Y. L. & Liu, F. A novel bispecific c-MET/CTLA-4 antibody targetting lung cancer stem cell-like cells with therapeutic potential in human non-small-cell lung cancer. *Biosci. Rep.* **39** (2019).

38 Xiong, M. *et al.* A Novel CD3/BCMA Bispecific T-cell Redirecting Antibody for the Treatment of Multiple Myeloma. *J. Immunother.* **45**, 78-88 (2022).

39 Bournazos, S., Gazumyan, A., Seaman, M. S., Nussenzweig, M. C. & Ravetch, J. V. Bispecific Anti-HIV-1 Antibodies with Enhanced Breadth and Potency. *Cell* **165**, 1609-1620 (2016).

40 Frei, J. C. *et al.* Bispecific Antibody Affords Complete Post-Exposure Protection of Mice from Both Ebola (Zaire) and Sudan Viruses. *Sci. Rep.* **6**, 19193 (2016).

41 Moshoette, T., Ali, S. A., Papathanasopoulos, M. A. & Killick, M. A. Engineering and characterising a novel, highly potent bispecific antibody iMab-CAP256 that targets HIV-1. *Retrovirology* **16**, 31 (2019).

42 Gupta, A. *et al.* Early Treatment for Covid-19 with SARS-CoV-2 Neutralizing Antibody Sotrovimab. *N. Engl. J. Med.* **385**, 1941-1950 (2021).

Figure. S1. Phylogeny, spike amino acid changes, and antibody evasion of the Omicron subvariant.

(a) Unrooted phylogenetic tree of Omicron and its subvariants along with other major SARS-CoV-2 variants, figure modified from the Nextstrain (https://nextstrain.org/ncov/gisaid/global/all-time), using data available from the GISAID initiative. (b) The structure of the closed prefusion conformation of the Omicron S trimer (PDB: 7TNW) is shown in the ribbon diagram with one protomer colored as NTD in orange, RBD in yellow, and the N-terminal segment of S2 in wheat. All mutations in the Omicron subvariant BA.1 and BA.2 are highlighted in the sphere model. (c) Specific spike mutations found in Omicron BA.2.12.1, Omicron BA.2.75 and Omicron BA4/5 are colored compared with the sequence of Omicron BA.2 sublineage (Orange, Omicron BA.2.12.1 specific mutations; Blue, Omicron BA.2.75 specific mutations; Violet, Omicron BA4/BA.5 specific mutations). (d, e) ELISA binding curves of three parental Omicron BA.1 RBD specific mAbs (d) and one parental SARS-CoV-2 RBD specific mAb (e) to Omicron sublineages RBD domains. The EC50 was determined by log (agonist) response of nonlinear regression and is displayed as the mean ± s.e.m. (f) Coomassie-stained SDS-PAGE analysis of five purified resultant bsAbs. The antibody samples are analyzed under reducing condition (+DTT).

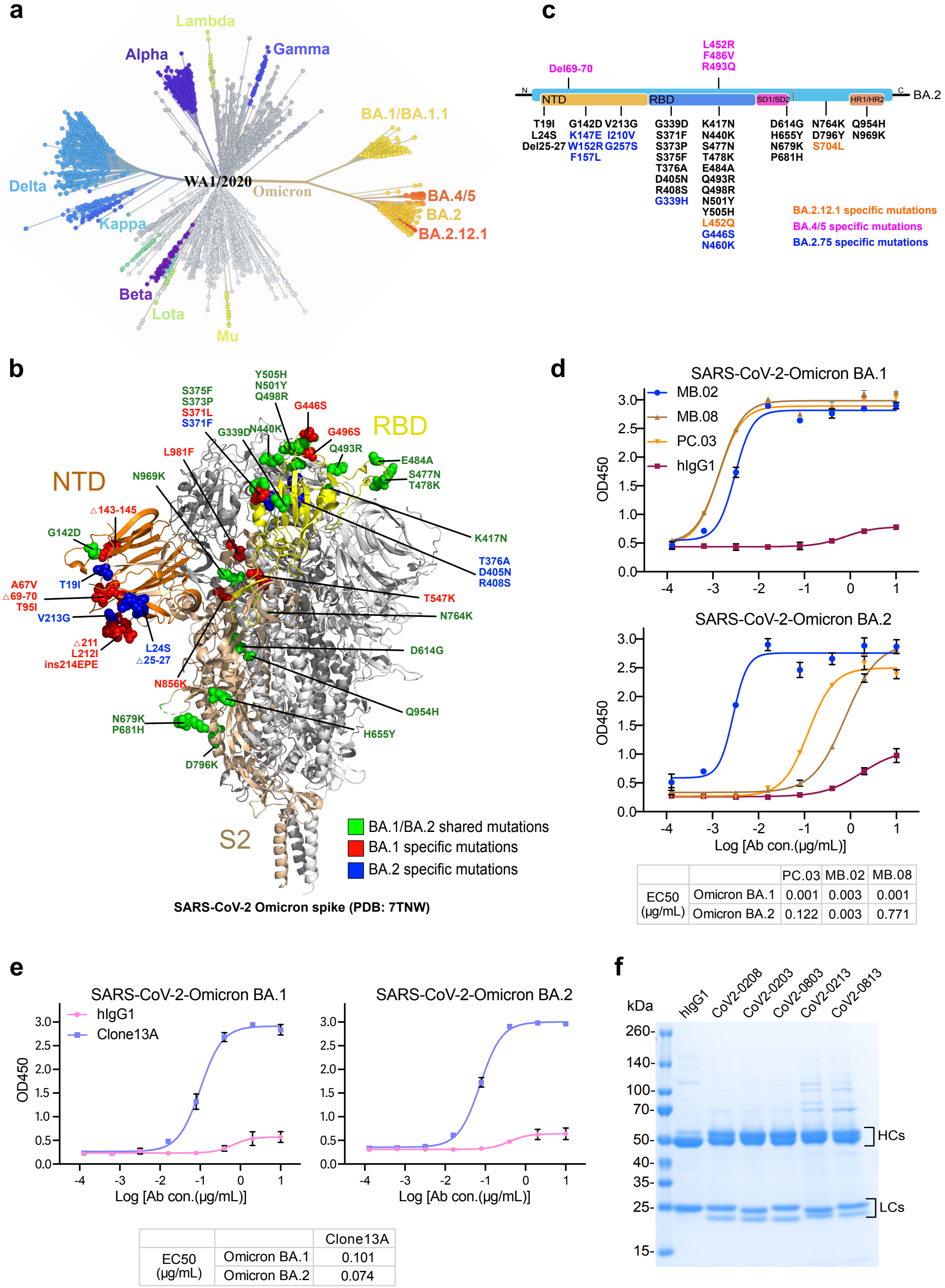

		PC.03	MB.02	MB.08
EC50 (μg/mL)	Omicron BA.1	0.001	0.003	0.001
	Omicron BA.2	0.122	0.003	0.771

		Clone13A
EC50 (μg/mL)	Omicron BA.1	0.101
	Omicron BA.2	0.074

Figure. S2. ELISA-based binding activities of resultant bsAbs are superior to those of its parental mAbs.

(a) ELISA binding curves of five resultant bsAbs with RBD proteins of SARS-CoV-2 WA-1, Delta, Omicron BA.1 and BA.2. The EC50 was determined by log (agonist) response of nonlinear regression and is displayed as the mean±s.e.m. (b) ELISA binding curves of two lead bsAbs and S309 with RBD proteins of SARS-CoV-2 WA-1, Delta, Omicron BA.1 and BA.2. The EC50 was determined by log (agonist) response of nonlinear regression and is displayed as the mean±s.e.m. (c) ELISA binding curves of CoV2-0213 with RBD proteins of MERS-CoV and SARS-CoV. The EC50 was determined by log (agonist) response of nonlinear regression and is displayed as the mean ± s.e.m.

a

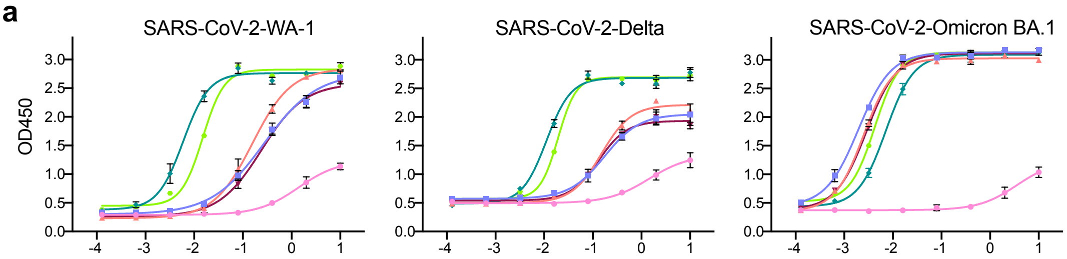

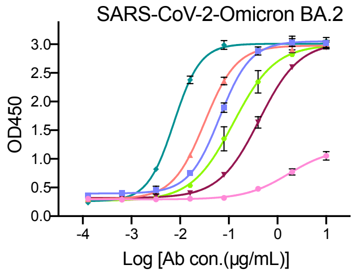

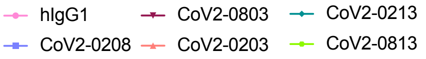

		CoV2-0208	CoV2-0203	CoV2-0803	CoV2-0213	CoV2-0813
EC50 (μg/mL)	WA-1	0.270	0.157	0.242	0.006	0.015
	Delta	0.174	0.150	0.131	0.011	0.019
	Omicron BA.1	0.002	0.003	0.003	0.007	0.004
	Omicron BA.2	0.065	0.032	0.423	0.007	0.125

b

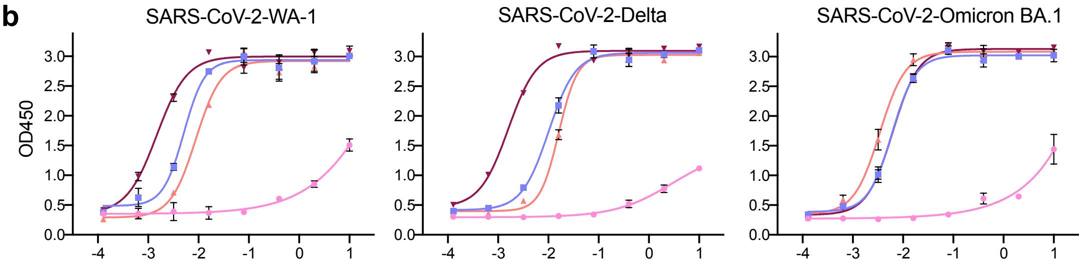

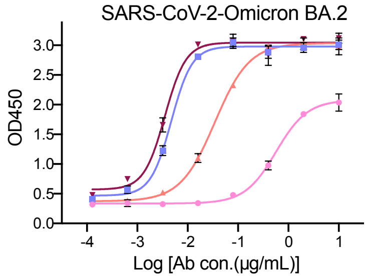

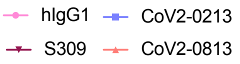

		CoV2-0213	CoV2-0813	S309
EC50 (μg/mL)	WA-1	0.005	0.009	0.002
	Delta	0.010	0.016	0.002
	Omicron BA.1	0.006	0.003	0.006
	Omicron BA.2	0.005	0.035	0.004

c

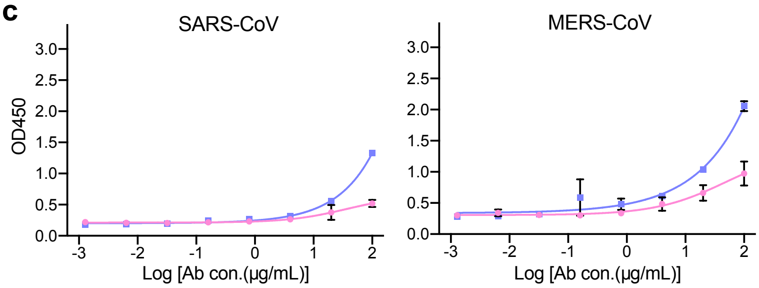

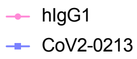

		CoV2-0213
EC50 (μg/mL)	SARS-CoV	> 50
	MERS-CoV	> 50

Figure. S3. ACE2/RBD binding inhibition for two lead bsAbs and flow gating strategy of neutralization assay.

(a) Competition ELISA of lead bispecific Abs with human ACE2 for epitope identification to SARS-CoV-2 WT, Delta, Omicron BA.1 and BA.2 RBD proteins. The data were obtained from a representative experiment with three replicates. Data are represented as mean ± s.e.m. IC50 values were calculated from ACE2 competition ELISA assays by fitting a log (inhibitor) response of a nonlinear regression model. (b) Representative flow gating plots.

a

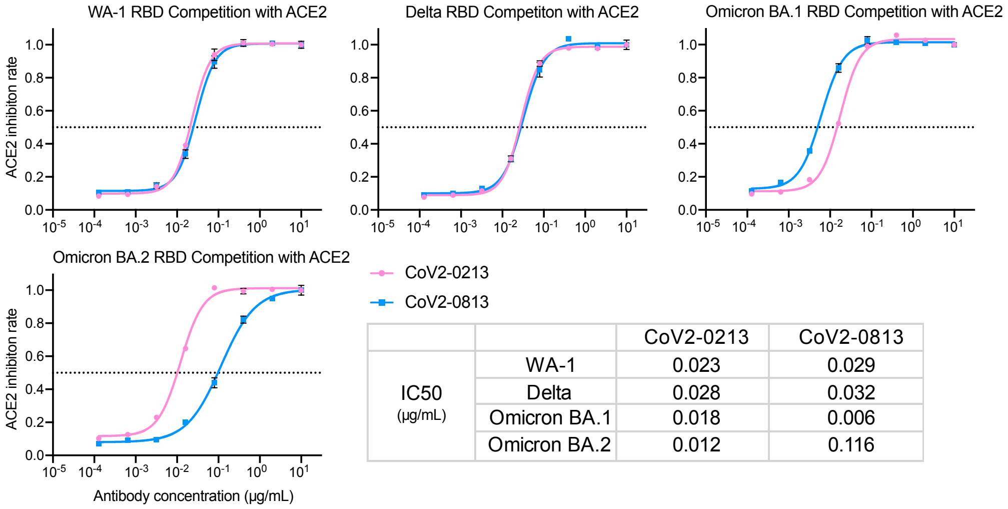

		CoV2-0213	CoV2-0813
IC50 (μg/mL)	WA-1	0.023	0.029
	Delta	0.028	0.032
	Omicron BA.1	0.018	0.006
	Omicron BA.2	0.012	0.116

b

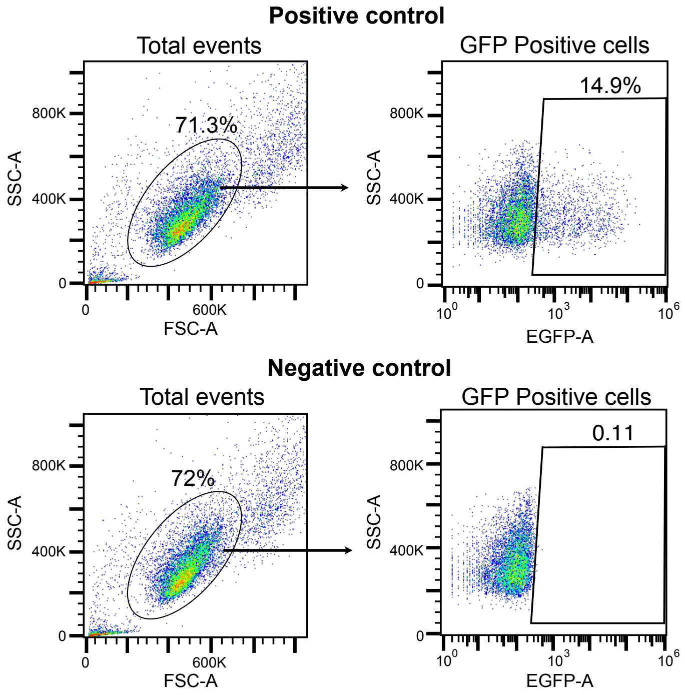

Figure. S4. Resistance of SARS-CoV and SARS-CoV-2 subvariants to neutralization by resultant bsAbs.

(a) Mutations of K440A, S446A and V486A on Omicron BA.1 RBD abolish CoV2-0213 neutralization. Serial dilutions of bsAb were added to test its neutralizing activity against indicated pseudotyped virus. The IC50 was determined by log (inhibitor) response of nonlinear regression and is displayed as the mean ± s.e.m. (b) Neutralization curves of remaining bsAbs against pseudotyped virus of ancestorial SARS-CoV-2 subvariants. Serial dilutions of bsAb were added to test its neutralizing activity against indicated pseudotyped virus. The IC50 was determined by log (inhibitor) response of nonlinear regression and is displayed as the mean ± s.e.m. (c) Structural analysis of mutational effects on binding of CoV2-0213 against emerging SARS-CoV-2 Omicron subvariants, BQ.1.1 and XBB.1. Site-directed mutagenesis was conducted in PyMOL.

a

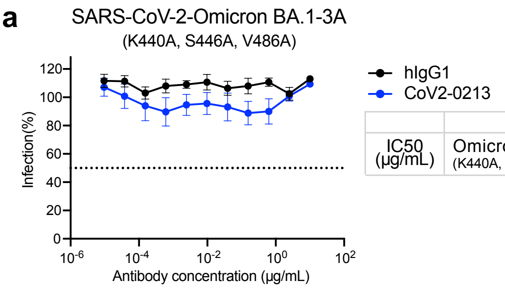

		CoV2-0213
IC50 (μg/mL)	Omicron BA.1-3A (K440A, S446A, V486A)	N/A

b

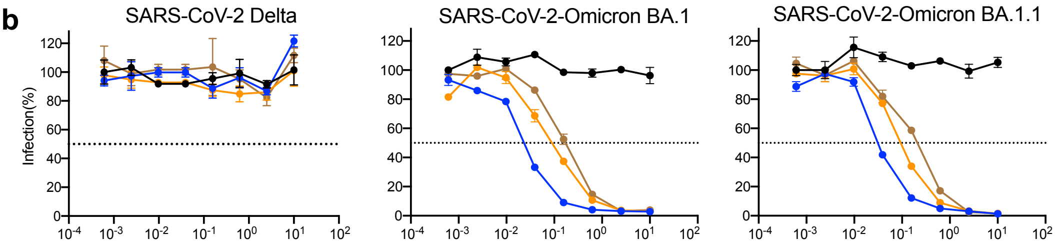

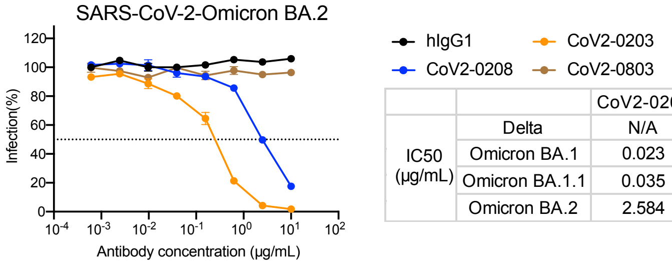

		CoV2-0208	CoV2-0203	CoV2-0803
IC50 (μg/mL)	Delta	N/A	N/A	N/A
	Omicron BA.1	0.023	0.091	0.169
	Omicron BA.1.1	0.035	0.101	0.188
	Omicron BA.2	2.584	0.210	N/A

c

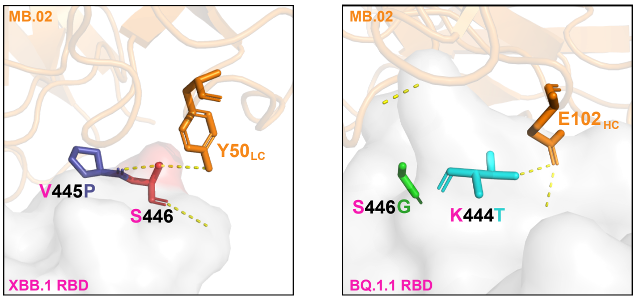

Figure. S5. Affinity of the RBDs of SARS-CoV-2 Omicron subvariants to CoV2-0213.

(a) Binding affinities of the RBDs of Omicron subvariant BA.1 and BA.1.1 to CoV2-0213 as measured by BLI. bsAbs were immobilized on an AHC sensor and tested for real-time association and dissociation of the RBD (BA.1, left panel, BA.1.1, right panel). Global fit curves are shown as red dotted lines. The vertical dashed lines indicate the transition between the association and dissociation phase. (b) Binding affinities of the RBDs of Omicron subvariant BA.2, BA.2.12.1, BA.3, and BA.4/5 to CoV2-0213 as measured by BLI. bsAbs were immobilized on an AHC sensor and tested for real-time association and dissociation of the RBD (BA.2, left upper panel, BA.2.12.1, right upper panel, BA.3, left lower panel, BA.4/5, right lower panel). Global fit curves are shown as red dotted lines. The vertical dashed lines indicate the transition between the association and dissociation phase.

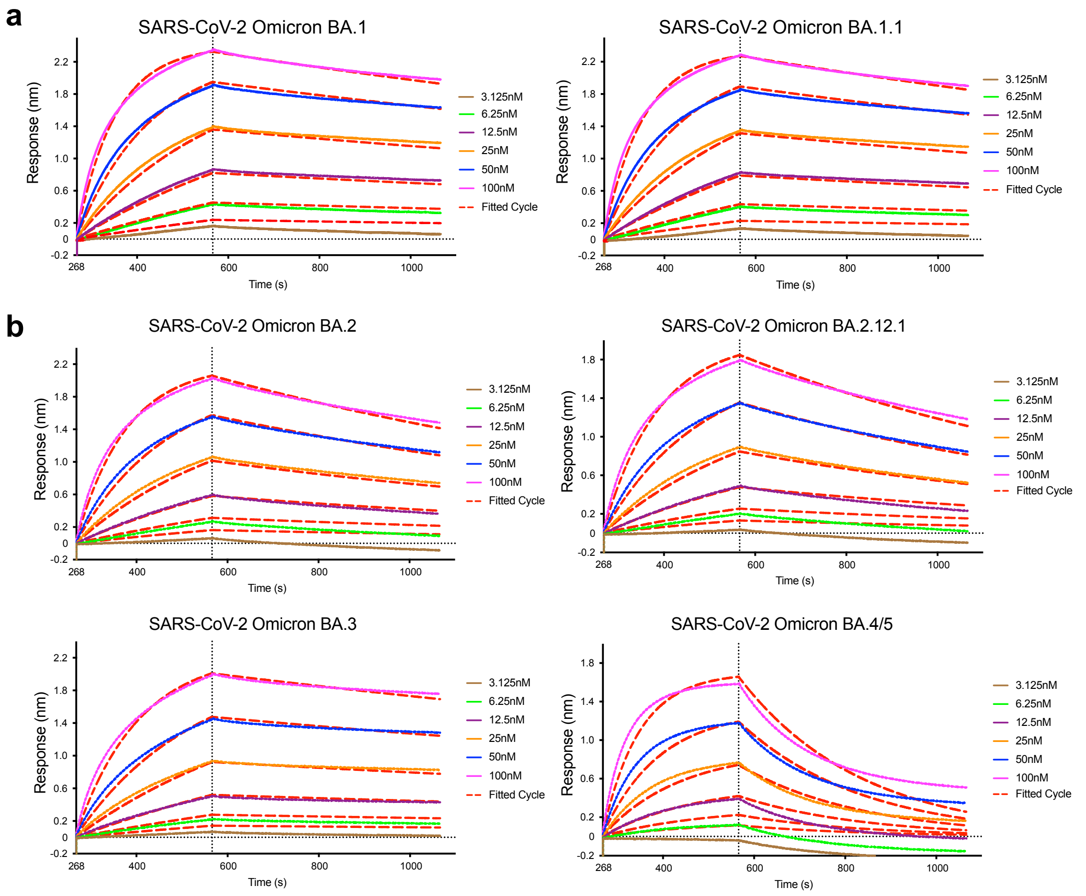

Figure. S6. Local flexibility and resolution estimation of the cryo-EM reconstructions.

(a) Local resolution estimation of the reconstructions of MB.02 Fab in complex with Omicron BA.1 spike trimer of two different conformations. (b) Example of one RBD-MB.02 Fab protomer that has flexible conformations. (c) Local resolution estimation of the reconstruction of bispecific antibody CoV2-0213 in complex with Omicron BA.5 spike trimer. (d) Overlay of Clone 13A and MB.02 onto the same down RBD with a neighboring up RBD in a trimeric spike indicates no steric hindrance.

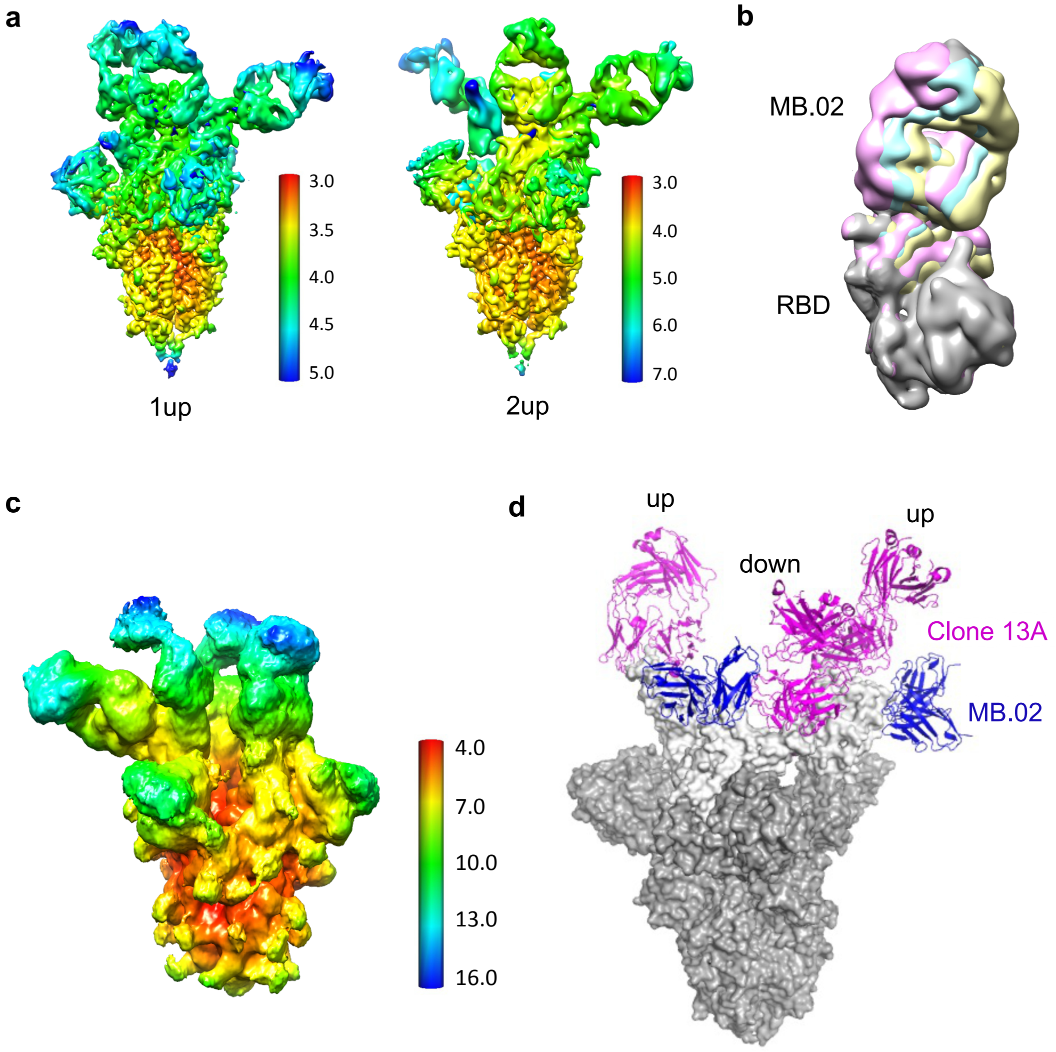

Figure. S7.

Cryo-EM data processing workflow of Omicron BA.1 spike in complex with MB.02 Fab (a) and omicron BA.5 spike in complex with bispecific antibody CoV-0213 (b).

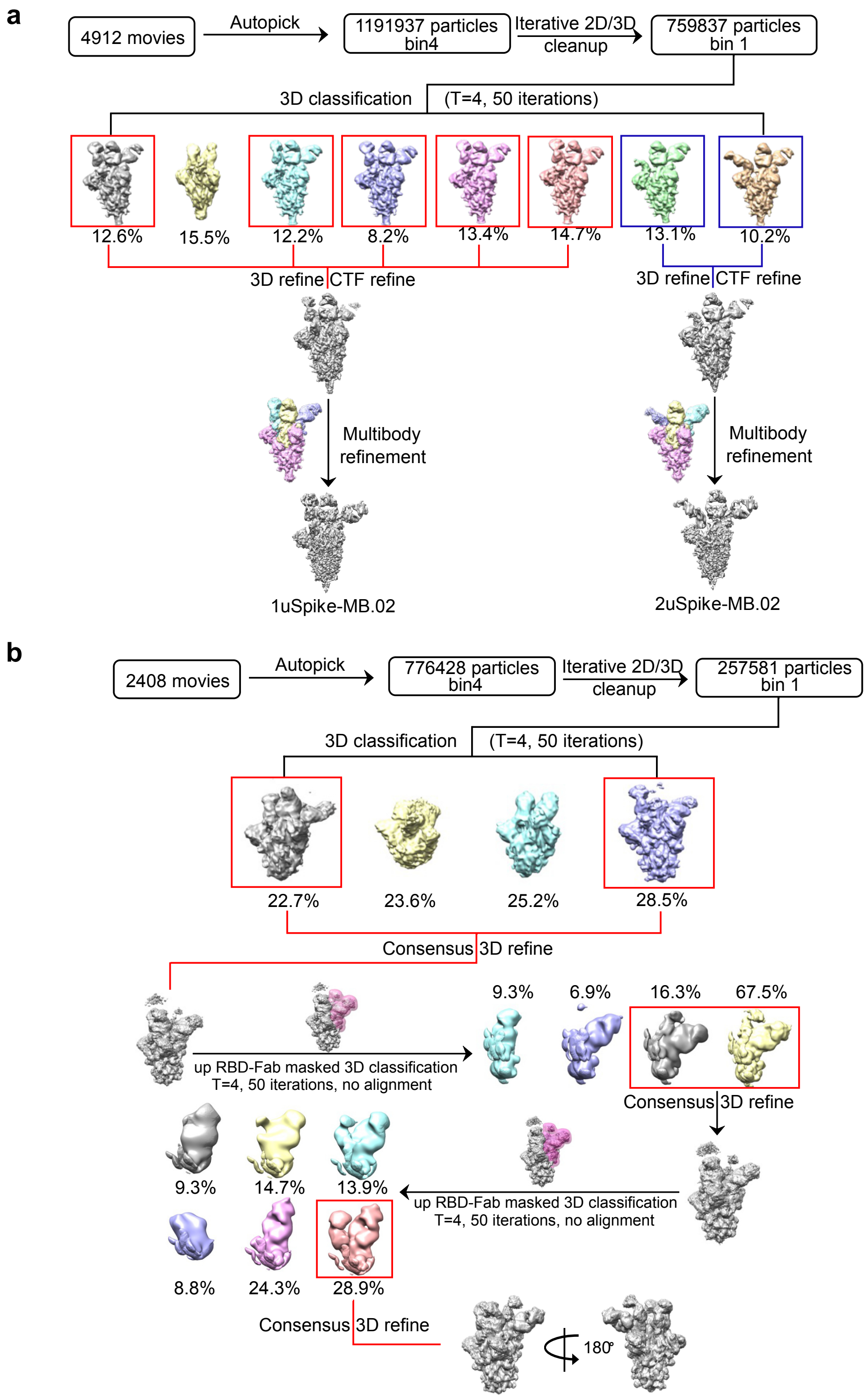

Figure. S8.

Superposition of the approval antibody structures onto the MB.02-bound RBD structure. Antibody Fabs are shown as ribbon diagrams, and the RBD is shown in surface representation. All the antibody structures were downloaded from the PDB (8DZH (MB.02), 7L7D (COV2-2196), 7L7E (COV2-2130), 6XDG (REGN10987), 6XDG (REGN10933), 7XCO (S309), 7MMO (LY-CoV1404), 7C01 (LY-CoV016), 7KMG (LY-CoV555)) for analysis. All the structural analysis figures were generated in PyMOL (Schrodinger, LLC).

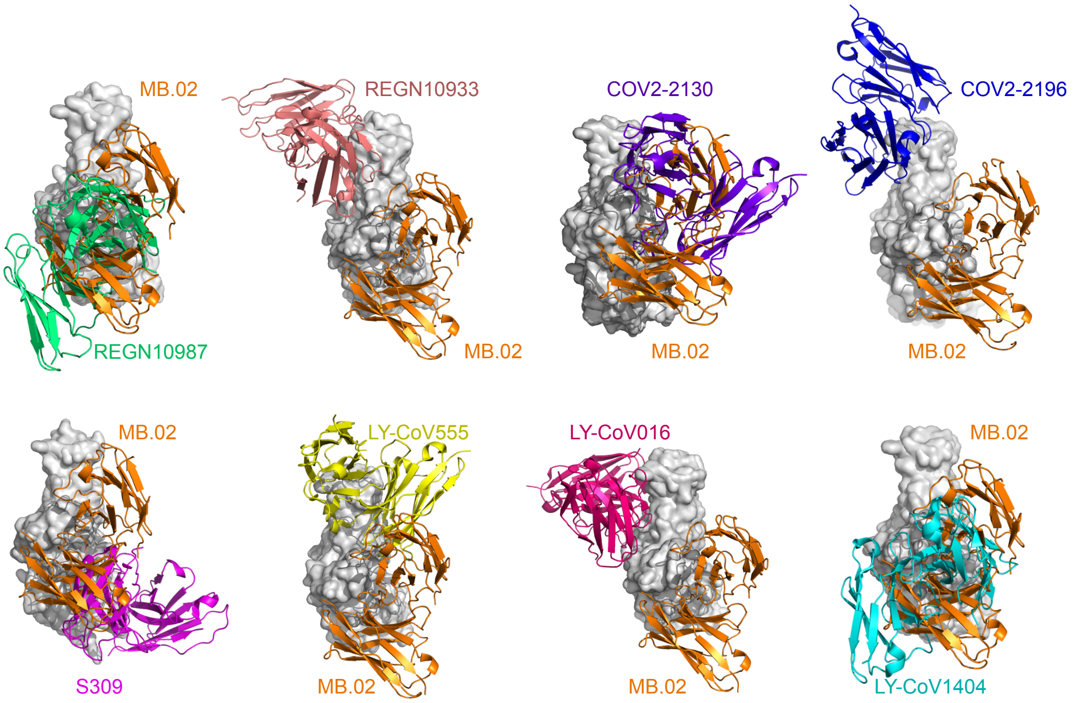

Table S1.

Cryo-EM Data Collection and Refinement Statistics

	1uSpike-MB.02<br>(PDB 8DZH)<br>(EMD-27798)<br><br>(MB.02::BA.1 Complex)	2uSpike-MB.02<br>(PDB 8DZI)<br>(EMD-27799)<br><br>(MB.02::BA.1 Complex)	1uSpike-CoV2-0213<br>(EMD-27800)<br><br><br>(CoV-0213::BA.5 Complex)
**Data collection and processing**			
Magnification	81000		
Voltage (kV)	300		
Electron exposure (e^-/Å^2)	64		64
Defocus range (μm)	-0.8 to -1.8	-0.8 to -1.8	-0.8 to -1.8
Pixel size (Å)	1.07		
Symmetry imposed	C1	C1	C1
Initial particle images (no.)	1191937		776428
Final particle images (no.)	464260	177042	26293
Map resolution (Å)<br>FSC threshold	3.2	3.5	7.7
**Refinement**			
Map sharpening *B* factor (Å^2)	-104 to -152	-110 to -253	0
Model map FSC (masked)	0.86	0.87	
Model composition<br>Non-hydrogen atoms<br>Protein residues<br>Ligands	 31967<br>3966<br>73	 31470<br>3918<br>63	
B factors (Å^2)<br>Protein<br>Ligand	 197<br>212	 234<br>227	
R.m.s. deviations<br>Bond lengths (Å)<br>Bond angles (°)	 0.01<br>0.9	 0.003<br>0.7	
Validation<br>MolProbity score<br>Clashscore<br>Poor rotamers (%)	 2.1<br>18.5<br>0.2	 2.0<br>18.3<br>0.4	
Ramachandran plot<br>Favored (%)<br>Allowed (%)<br>Disallowed (%)	 94.5<br>5.3<br>0.2	 96.6<br>3.3<br>0.2	

*1uSpike-MB.02: Omicron BA.1.1.529 Spike trimer with 2 RBDs down in complex with MB.02 Fab; 2uSpike-MB.02: Spike trimer with 1 RBD down in complex with MB.02 Fab; 1uSpike-CoV2-0213: Omicron BA.5 Spike trimer with 2 RBDs down in complex with bispecific antibody CoV2-0213.
